# Applying the New World Kirkpatrick Model to evaluate an online training course introducing a multi-sensory device

**DOI:** 10.1093/geroni/igab046.2827

**Published:** 2021-12-17

**Authors:** Jennifer Perion, Victoria Steiner, Jennifer Kinney, Kimberly McBride, Barbara Saltzman

**Affiliations:** 1 University of Toledo, Temperance, Michigan, United States; 2 University of Toledo, Toledo, Ohio, United States; 3 Miami University, Oxford, Ohio, United States; 4 University of Toledo, University of Toledo, Ohio, United States

## Abstract

Online education offers care providers flexibility and convenience. Applying the New World Kirkpatrick Model of training evaluation, this descriptive study evaluated the design and content of a 30-40 minute online training course that introduces direct-care workers to a multi-sensory device to help manage dementia symptoms in older adults. Following course completion, an online survey obtained ratings of engagement (i.e., aesthetics, ease of use, novelty, and involvement), relevance, and knowledge/skills gained from the training using a 5-point Likert scale. A convenience sample of 72 undergraduate students enrolled in health science and human service programs at a Midwestern university participated. The majority were white (83.3%), non-Hispanic (81.9%) females (88.9%). Most participants agreed or strongly agreed (median=4) with positive statements related to engagement with the course. Statements about relevance to their intended career were rated even higher (median=5). Wilcoxon signed-rank tests for matched pairs revealed statistically significant improvements on self-reported pre-post knowledge/skills scores (p<0.005). The results indicated that participants found the training aesthetically pleasing, easy to use, novel, and that it encouraged user involvement. Participants thought the topics covered were relevant to the professional career they are pursing, and they learned new knowledge/skills. Responses to open-ended questions suggested improvements to the design (e.g., color choice) and content (e.g., expanded topics and resources). Future research will evaluate a revised course with direct-care workers who will use the multi-sensory device in long term care facilities. Subsequently, an intervention study will determine the effectiveness of the device in increasing the well-being of people with dementia.

